# Association of familial Mediterranean fever and epicardial adipose tissue: A systematic review and meta‐analysis

**DOI:** 10.1002/hsr2.693

**Published:** 2022-06-13

**Authors:** Karam R. Motawea, Omneya A. Kandil, Joseph Varney, Merna Aboelenein, Nancy Ibrahim, Ahmed Shaheen, Lina T. Khairy, Agyad Bakkour, Ali H. H. Muwaili, Dhuha H. H. Muwaili, Fatima A. A. Abdelmajid, Eman M. S. Ahmad, Mhd K. Albuni, Elias Battikh, Bisher Sawaf, Sarya Swed, Safaa M. A. Ahmed, Dina M. Awad, Jaffer Shah, Hani Aiash

**Affiliations:** ^1^ Faculty of Medicine Alexandria University Alexandria Egypt; ^2^ School of Medicine American University of the Caribbean Cupecoy Sint Maarten; ^3^ Faculty of Medicine The National Ribat University Al‐Ribat Sudan; ^4^ Faculty of Medicine Albaath University Homs Syria; ^5^ Faculty of Medicine Ivano‐Frankivsk National Medical University Ivano‐Frankivsk Ukraine; ^6^ Faculty of Medicine University of Medical Sciences and Technology Khartoum Sudan; ^7^ Departments of Obstetrics and Gynecology Nile Valley University Atbra Sudan; ^8^ Department of Internal Medicine Hamad Medical Corporation Doha Qatar; ^9^ Faculty of Medicine Aleppo University Aleppo Syria; ^10^ Faculty of Medicine Shendi University Shendi Sudan; ^11^ Medical Research Center Kateb University Kabul Afghanistan; ^12^ Cardiovascular perfusion Department Upstate Medical University Syracuse New York USA; ^13^ Family Medicine Department Suez Canal University Ismailia Governorate Egypt

**Keywords:** epicardial adipose tissue, familial Mediterranean fever, lipids, Meta analysis

## Abstract

**Background and Aim:**

Some studies reported a positive link between familial Mediterranean fever (FMF) and epicardial adipose tissue. Our meta‐analysis aimed to evaluate whether there is a significant association between FMF and increased epicardial adipose tissue thickness.

**Methods:**

We searched the following databases: PUBMED, WOS, OVID, SCOPUS, and EMBASE. Inclusion criteria were any original articles that reported epicardial adipose tissue in FMF patients with no age restriction, excluding reviews, case reports, editorials, animal studies, and non‐English studies. Thirty eligible studies were screened full text but only five studies were suitable. We used RevMan software (5.4) for the meta‐analysis.

**Results:**

The total number of patients included in the meta‐analysis in the FMF patients group is 256 (mean age = 24.3), and the total number in the control group is 188 (mean age = 24.98). The pooled analysis between FMF patients and controls was [mean difference = 0.82 (95% CI = 0.25–1.39), *p*‐value = 0.005]. We observed heterogeneity that was not solved by random effects (*p* > 0.00001). We performed leave one out test by removing the Kozan et al. study, and the heterogeneity was solved (*p* = 0.07), and the results were (MD = 0.98, 95% CI = 0.52–1.43, *p*‐value < 0.0001).

**Conclusion:**

FMF patients are at increased risk of developing epicardial adipose tissue compared to controls. More multicenter studies with higher sample sizes are needed to support our results.

## 1 INTRODUCTION

Familial Mediterranean fever (FMF) is characterized by intermittent attacks of fever, painful inflammation, arthritis, and abdominal pain that last from few hours to days and recur after weeks or months. It is an autosomal recessive illness seen mainly in Turkish, Armenian, and Mediterranean region ethnics and diagnosed by Tel‐Hashomer clinical criteria, consisting of two or more significant symptoms (from febrile episodes with serositis or a favorable response to colchicine or amyloidosis) or one major plus two minor symptoms (a first degree relative with FMF, erysipelas like erythema and recurrent febrile episodes).^1^

FMF increases the risk of developing coronary artery diseases (CAD), as witnessed in several studies that reported a higher prevalence of CAD in FMF patients.^2^, ^3^ The nonsubsiding inflammation that occurs in FMF is a critical player in the pathogenesis of plaque formation and blood vessels thickening, explaining this higher incidence of CAD.^4^ Yet abnormal lipid profile that increases atherosclerosis risk due to high Triglycerides/high density lipoprotein (TG/HDL) ratio is not always associated with an increased carotid intima‐media thickness. Thus, carotid intima‐media thickness can not detect subclinical atherosclerosis. Several studies failed to show any significant difference in carotid intima‐media thickness (CIMT) between FMF patients and other patients despite an increase in cholesterol and TG levels.^5^, ^6^

On the other hand, some studies have found the Epicardial adipose thickness had a positive correlation with cholesterol and TG levels and thus could be used for early detection of subclinical atherosclerosis. The study aims to find out if there is an association between FMF patients and epicardial adipose tissue and if epicardial adipose tissue could be used as an early predictor of atherosclerosis in FMF patients.^7^

## 2 METHODS

### 2.1 *Search and identification of studies

We searched the following databases: PUBMED, WOS, OVID, SCOPUS, and EMBASE through June 2021. We also searched Open Grey, Lilacs, and Proquest databases for relevant literature and dissertations. Search terms used were (“Familial Mediterranean Fever” OR “Familial Paroxysmal Polyserositis” OR “Periodic Disease” OR “Periodic Peritonitis” OR “Recurrent Polyserositis”) AND (“epicardial adipose tissue” OR “lipids”). Detailed search strategy for each database and the date, which the database was last consulted are available in Supporting Information.

### 2.2 *Selection process and inclusion criteria

Yielded results from databases were imported into Covidence.^8^

From the searches, initial screening of title and abstract was done along with retrieval of potentially relevant references by four authors. Next, full‐text studies were retrieved, if there is a conflict among the authors who screened the studies, a final decision by the first author was taken. The full‐text screening was performed for the papers using predetermined inclusion criteria: any controlled clinical trials, case control studies, controlled retrospective or prospective cohort studies that reported epicardial adipose tissue thickness in FMF patients and controls without FMF with no age restriction excluding reviews, case reports, editorials, animal studies, and non‐English studies. These controlled studies were included in the analysis if they measured epicardial adipose tissue thickness in FMF patients and controls without FMF.

### 2.3 Data extraction and quality assessment

Data were extracted from each study by two authors after obtaining the full paper into excel sheets. Each author revised the work of the other one, if there is a conflict between the authors who extracted the data, a final decision by the first author was taken. Quality assessment was done by the New castle Ottawa scale assessment tool. The studies were ranked as good, fair, or poor.

### 2.4 Statistical analysis

A meta‐analysis was carried out evaluating the association between FMF patients and epicardial adipose tissue.

RevMan 5.4 was used for statistical analysis. The continuous outcomes were measured as mean difference (MD) and standard deviation (SD) with a 95% confidence interval. If heterogeneity (Chi‐square *p*‐value < 0.05) was observed, a random effect model was used otherwise, a fixed‐effect model was performed. The results were considered significant if the *p*‐value was less than 0.05.

## 3 RESULTS

**Figure 1:**
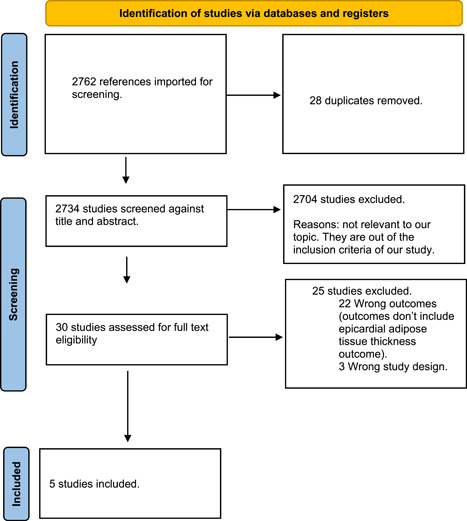
The Prefered Reporting Items for Systematic Revie and Meta Analysis (PRISMA) flow diagram

After a search of the literature, 2762 papers resulted and became 2734 eligible for the title and abstract screening after removal of duplicates. Of 2734, 2704 were irrelevant, and 30 were eligible for full‐text screening. There were 25 studies that might appear to meet the inclusion criteria, but were excluded as studies^9^, ^10^, ^11^, ^12^, ^13^ because of wrong study design and wrong outcomes. Five studies were included in the meta‐analysis after performing full‐text screening, as shown in (Figure 1). We aimed to pool the data in the five studies to find an association between the increased thickness of epicardial adipose tissue and FMF patients. The quality assessment and summary of the included studies are shown in Tables 1 and 2, respectively. The overall quality was high in the included studies.

**Table 1:** Quality assessment

ID	Newcastle Ottawa scales to AHRQ standards			Rating	Comments		
	Selection	Comparability	Exposure		Selection	Comparability	Exposure
Uluca, 2015	2 stars	1 star	1 star	Fair quality	1—adequate definition of cases, 2— definition of control	Comparable in age and gender only (other factors not clear)	1—patients diagnosed according to Tel‐Hashomer criteria, 2—not clear if controls were assesed by the same method, 3— responsiveness not described
Ghobrial, 2020	4 stars	1 star	2 star	Good quality	1—adequate definition of cases, 2—cases representitive 3—community controls 4— definition of control	Comparable in age and gender only (other factors not clear)	1—patients diagnosed according to Tel‐Hashomer criteria, 2— controls were assesed by the same method, 3— responsiveness not described
Kozan, 2019	2 stars	1 star	1 star	Fair quality	1—adequate definition of cases, 2— definition of control	Comparable in age and gender only (other factors not clear)	1—patients diagnosed according to Tel‐Hashomer criteria, 2—not clear if controls were assesed by the same method, 3— responsiveness not described
Kucuk, 2013	3 stars	1 star	1 star	Fair to good quality	1—adequate definition of cases, 2—cases representitive, 3—no description of selection of controls, 4— definition of control	Comparable in age and gender only (other factors not clear)	1—patients diagnosed according to Tel‐Hashomer criteria, 2—not clear if controls were assesed by the same method, 3— responsiveness not described
Kirbas, 2016	4 stars	1	2	Good quality	1—adequate definition of cases, 2—cases representitive 3—controls were selected from the same sample as the cases 4—definition of control	Comparable in age, gender, and ethnicity	1—patients diagnosed according to Tel‐Hashomer criteria, 2—controls were assesed by the same method, 3— responsiveness not described

**Table 2:** Summary of the included studies

ID	Gender (M/F)	Age (years, mean, SD)	Design	Duration of the disease (mean, SD)	Study arms	Endpoints (outcomes)	Conclusion
Uluca, 2015	Cases:19/26 _ control 24/30	Cases: 8.1 (4.1)/control: 7.9 (4.6)	Case‐control study	4.1 (3.0)	Forty‐five familial Mediterranean fever (FMF) patients diagnosed according to Tel‐Hashomer criteria and age‐ and gender‐matched 54 healthy normal weighted controls were enrolled into the study. Patients who experienced an FMF attack within 2 weeks before admission, overweighed children, and cases with dyslipidemia or any coexistent inflammatory disease were excluded from the study	Epicardial adipose tissue thickness of the children with FMF were found to be significantly greater than that of controls (5.1 ± 1.4 vs. 4.5 ± 0.9 mm, *p* = 0.036). FMF patients had significantly higher MPV values compared with the controls (7.8 ± 1.1 vs .7.3 ± 1.4 fl, *p *= 0.044). Age at diagnosis, duration of delay in diagnosis, and MPV values were found to be correlated with EAT thickness in the patient group (*r *= 0.49, *p *= 0.001 for the former parameters and *r *= 0.32, *p *= 0.04 for MPV).	Epicardial adipose tissue thickness and MPV values seem to be increased in children with FMF. These findings may indicate an increased risk of atherosclerosis in FMF patients.
Ghobrial, 2019	_	Cases: 10.4 (2.4)/control:11.1 (2.6)	Case‐control study	6.8 (3.2)	Study carried on 30 FMF children diagnosed clinically according to Tel‐Hashmomer criteria. It also included 30 healthy children coming for follow‐up visits at the outpatient general clinic.	EAT in patients' group was significantly greater than that of controls (5.2162.3 vs. 2.8162.96 mm, *p* = 0.001) and was correlated with cholesterol level and platelets count (*p* = 0.047 and 0.018, respectively).	This study concluded that EAT thickness was statistically increased in FMF patients than controls with a positive correlation with cholesterol level and platelet count. These findings suggest a higher risk for atherosclerosis in these patients. Follow‐up study is needed to verify the effect of treatment of FMF on the EAT thickness. Further studies with larger number of patients following‐up EAT are needed to verify this finding.
Kozan, 2019	Cases/control :65/38	Cases: 37.3 (12.7)/control: 35.5 (9.8)	Prospective, cross‐sectional study	_	Sixty‐five patients diagnosed with FMF on the basis of Tel‐Hashomer criteria, 11 using colchicine, and 38 healthy individuals between the ages of 18–70.	The FMF patients had significantly higher levels of CRP, epicardial adipose tissue, and pulse velocity (*p* < 0.001, <0.05, <0.005, respectively) as compared with the control group. However, the serum vitamin D levels in the two groups were observed o be similar (*p* = 0.486). weak but significant positive correlations were observed between epicardial adipose tissue thickness and C‐reactive protein (*r* = 0.302, *p* < 0.005), epicardial adipose tissue thickness and pulse velocity (*r* = 0.263, *p* < 0.01), and C‐reactive protein and pulse velocity (*r* = 0.235, *p* < 0.05).	Thickness of epicardial adipose tissue and pulse velocity are observed to increase in FMF patients
Kirbas, 2016	Cases (females only): 37/control (females only): 40	Cases: 27.3 (5.1)/contol: 28.9 (2.4)	Case‐control study	5.2 (2.4)	Forty‐five consecutive pregnant women with FMF and 50 healthy women with uncomplicated pregnancy (as the control group), all in the third trimester and matched for maternal and gestational ages, were recruited between January 2014 and july 2015	No differences in Pd and corrected QT values were found between the groups. Epi cardial fat thickness values were significantly higher in the FMF group compared with the control group (*p* = 0.015). A positive correlation was found between FMF duration and epicardial fat thickness (*r* = 0.350, *p* = 0.042)	Pd, a noninvasive marker of potential atrial arrhythmia and QT‐d, a noninvasive marker of potentially lethal ventricular tachyarrhythmia, constitute a recent contribution to the field of noninvasive electrocardiology. Pd and QT‐d values were not altered in pregnant women with FMF who already put on colchicine treatment, with no increased risk of atrial or ventricular arrhythmias indicated. Colchicine may have a cardio‐protective effect beyond the effect mediated through suppression of inflammation
Kucuk, 2013	Cases/control :79/26	Cases: 38.4 (10)/control: 41.5 (9.5)	Case‐control study	_	Seventy‐nine FMF patients who had been diagnosed according to Tel‐Hashomer criteria in Rheumatology outpatient clinic of a university hospital were included in the study between May 2012 and December 2012. Twenty‐six age and sex‐matched healthy individuals were recruited as the control group.	The EAT was thicker in patients with FMF than the control group (EAT 0.47 ± 0.13 vs. 0.36 ± 0.10 cm, *p* = 0.001). Carotid intima‐media thickness (CIMT) was also greater in patients with FMF than the control group (0.78 ± 0.2 vs. 0.68 ± 0.13 mm, *p* = 0.24). In correlation analysis, EAT was correlated with CIMT (*r* = 0.17, *p* = 0.1), CRP (*r* = 0.31, *p* = 0.005), BMI (*r* = 0.14, *p* = 0.16), total cholesterol (*r* = 0.170, *p* = 0.086), LDL (*r* = 0.212, *p* = 0.035) and age (*r* = 0.188, *p* = 0.054). CIMT was correlated with CRP (*r* = 0.211, *p* = 0.05), serum creatinine (*r* = 0.224, *p* = 0.022), total cholesterol (*r* = 0.231, *p* = 0.019), LDL (*r* = 0.219, *p* = 0.03), triglyceride (*r* = 0.214, *p* = 0.03), age (*r* = 0.453, *p* < 0.001), serum glucose (*r* = 0.267, *p* = 0.022) and BMI (*r* = 0.241, *p* = 0.013). In multivariate linear regression model, total cholesterol level (β: 0.399, t:2.716), CRP (β: 0.150, t: 2.139) and BMI (β: 0.431, t: 2.581) were found to be independent predictors of EAT.	Both EAT and CIMT were significantly greater in FMF patients than control subjects. In patients with FMF, EAT may be a novel marker of increased cardiovascular risk

CRP, C‐reactive protein; LDL, low dose lipoprotien; MPV, mean platelets volume.

### 3.1 Analyses

The total number of patients included in the meta‐analysis in the FMF patients group is 256 (mean age = 24.3), and the total number in the control group is 188 (mean age = 24.98).

**Figure 2:**
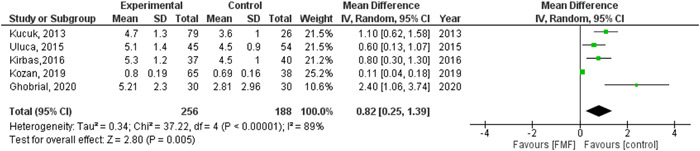
Forest plot of the association between familial Mediterranean fever and epicardial adipose tissue

The pooled analysis between FMF patients and controls was (MD = 0.82, 95% CI = 0.25–1.39, *p*‐value = 0.005), we observed heterogeneity that was not solved by random effects (*p* > 0.00001), as shown in Figure 2.

**Figure 3:**
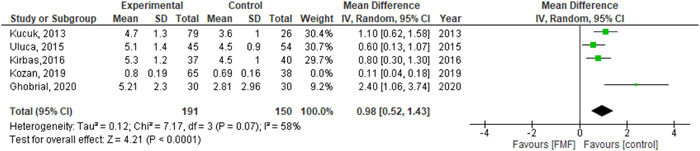
Forest plot of the association between FMF and epicardial adipose tissue after performing leave‐one‐out test

We performed leave one out test by removing (Kozan, 2019) study and the heterogeneity was solved (*p* = 0.07) and the results were (MD = 0.98, 95% CI = 0.52–1.43, *p*‐value < 0.0001), as shown in Figure 3.

**Figure 4:**
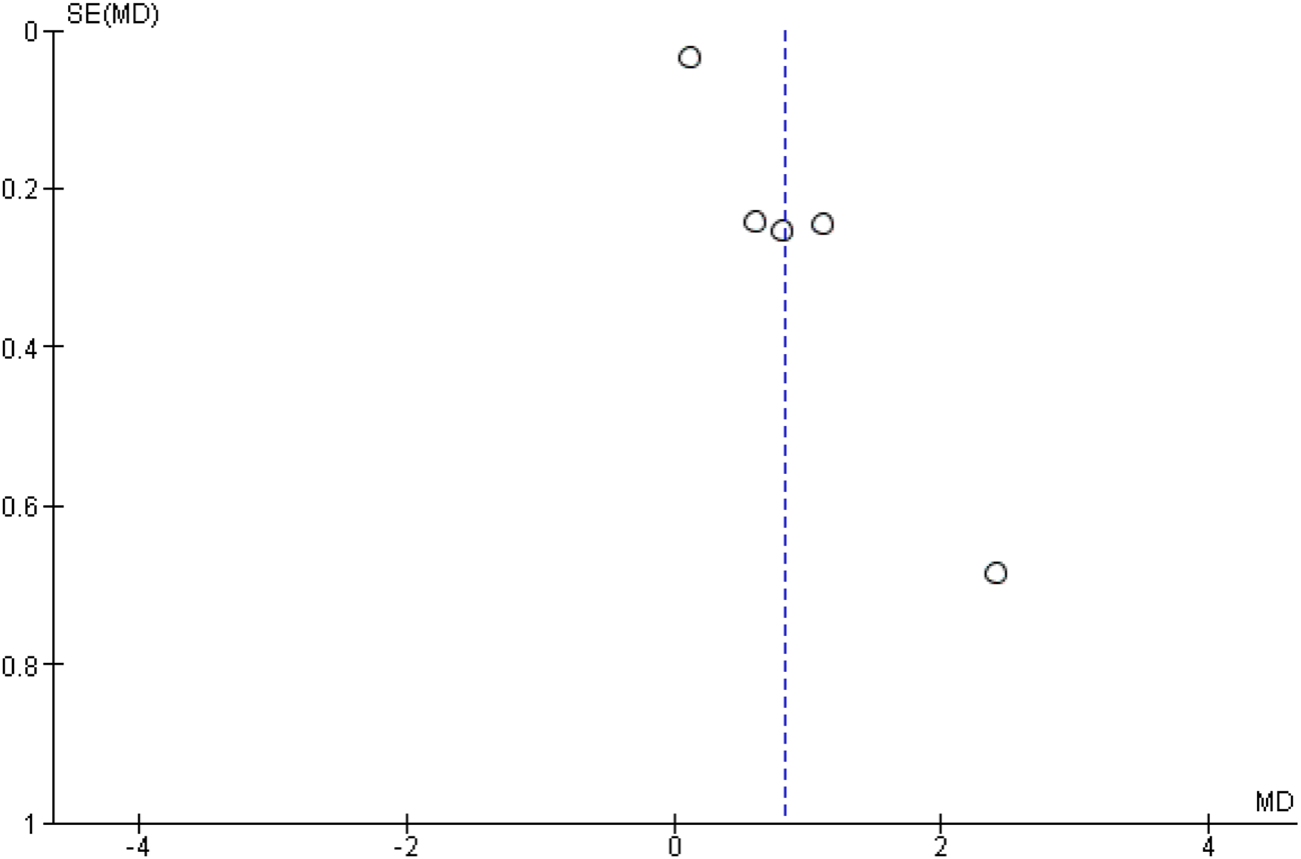
Publication bias

No publication bias was observed among included studies, as shown in Figure 4.

## 4 DISCUSSION

A statistically significant association was found between FMF and increased epicardial adipose tissue thickness compared to controls.

Epicardial adipose tissue (EAT) surrounds the myocardium and is primarily made up of adipocytes. Stromal, nerve, vascular, and even inflammatory cells are also known to exist in EAT.^8^ EAT is known to have less adipocyte density than other visceral fat stores, with increased efficiency in fatty acid intake and secretion.^14^ EAT is part of the visceral adipose tissue between the heart and pericardium, including both atrioventricular and interventricular sulcus, and is situated around the coronary arteries.^15^

Transthoracic echocardiography is typically utilized to visualize the EAT, which is depicted as a thick line above the right ventricles free wall.^16^

The EAT is thicker with increased volume in patients with obesity, impaired glucose tolerance, metabolic syndrome, hypertension, diabetes, and atherosclerosis.^17^, ^18^

The expression and secretion of multiple interleukins (IL‐1, ‐1β, ‐6, ‐8, and ‐10) are increased from EAT in patients with coronary artery disease versus the healthy control group.^19^ Furthermore, EAT is also known to secrete higher levels of reactive oxygen species than subcutaneous fat tissue in those who have coronary artery disease.^20^ The utility of epicardial adipose tissue thickness and mean platelet volume are relatively new dimensions used to assess the risk of atherosclerosis.^21^, ^22^, ^23^ FMF is thought to predispose patients to atherosclerosis due to its inflammatory process.^24^, ^25^

FMF is directly associated with nod‐like receptor family pyrin domain‐containing three inflammasome (NLRP3) dysfunction, which leads to IL‐1β dependent auto‐inflammation.^26^ The cytokines IL‐6 and TNF‐α were also increased in FMF patients both during attacks and in the attack‐free periods.^27^ The inflammation leads to decreased aortic elasticity, increased arterial stiffness,^28^ vascular dysfunction,^29^ as well as pericarditis, and even rhythm disorders.^30^ This systemic inflammation eventually leads to atherosclerosis and cardiovascular disease in FMF, a significant cause of mortality.^31^

A recent study stated a theory that suspect a positive relationship between FMF and EAT, as the cytokine profiles of EAT and FMF disease have a similarity which may have some roles in thickening of EAT in FMF patients.^7^ Our study found a statistically significant association between FMF and increased epicardial adipose tissue thickness compared to controls.

Currently, few studies have assessed the relationship between EAT thickness in FMF patients. Uluca et al. conducted a study of 45 FMF children against a control group and found that FMF children have increased EAT.^7^

Patients' age at diagnosis, duration of delay in diagnosis, and mean platelets volume (MPV) values correlated with EAT thickness as well.^7^ Ghobrial conducted another study on 30 6–18‐year‐old children and found a significant correlation between EAT thickness in all heart chambers and platelet count in FMF cases.^32^ No correlation between the age of FMF diagnosis or age at disease onset and EAT thickness.^32^ Kozan et al. tested 65 FMF patients against control and found the EAT thickness increased in the diseased group. Other parameters were associated with cardiovascular risk Vitamin D deficiency and pulse wave velocity increases.^33^ Kucuk et al. conducted a most recent study of 79 FMF patients and found similar findings that EAT thickness increased in these patients compared to controls.^34^

Regarding the main findings of this current review that determine the existence of significant association between FMF and EAT, these outcomes may need a strict follow‐up and the implementation of appropriate preventative measures. It is essential to determine if FMF patients have a higher risk of atherosclerosis, even when they're young. Measurement of mean platelet volume and cholesterol level are easy and inexpensive tests for investigation of thrombosis and atherosclerosis in FMF patients, also echocardiography is an easy and noninvasive way that could be used to measure EAT thickness.^32^, ^35^

The results of this study may be interpreted in clinical practice, as epicardial adipose tissue thickness may be used as a marker to assess the risk of atherosclerosis in FMF patients. FMF patients should perform echocardiography regularly to measure EAT, as they are at increased risk of developing increased EAT. Future clinical trials using therapies targeting EAT in FMF patients may be useful in primary prevention of EAT and atherosclerosis in high‐risk patients.

Our study is limited by few numbers of studies and patients included, only case control and cohort studies included, as we found no published randomized control trials about the topic and the observed heterogeneity among studies due to different study designs, so further randomized clinical trials with higher numbers of patients are needed to support our findings.

## 5 CONCLUSION

Our meta‐analysis revealed that Familial Mediterranean Fever patients are at elevated risk of developing increased epicardial adipose tissue thickness. Epicardial adipose tissue is important in predicting atherosclerosis in FMF patients, as they are at increased risk of developing atherosclerosis. More multicenter studies with higher sample sizes are needed to support our findings.

## AUTHOR CONTRIBUTIONS

**Karam R. Motawea**: Conceptualization; formal analysis; investigation; methodology; project administration; supervision; writing—original draft; writing—review and editing. **Omneya A. Kandil**: Data curation; writing—original draft; writing—review and editing. **Joseph Varney**: Investigation; writing—original draft; writing—review and editing. **Merna Aboelenein**: Data curation; writing—original draft. **Nancy Ibrahim**: Data curation; writing—original draft. **Ahmed Shaheen**: Writing—review and editing. **Lina T. Khairy**: Data curation; writing—original draft. **Agyad Bakkour**: Writing—original draft; writing—review and editing. **Ali H. H. Muwaili**: Writing—review and editing. **Dhuha H. H. Muwaili**: Writing—original draft. **Fatima A. A. Abdelmajid**: Writing—original draft; writing—review and editing. **Mhd K. Albuni**: Writing—original draft; writing—review and editing. **Elias Battikh**: Writing—original draft; writing—review and editing. **Bisher Sawaf**: Visualization; writing—original draft. **Sarya Swed**: Writing—review and editing. **Safaa M. A. Ahmed**: Writing—original draft. **Dina M. Awad**: Data curation; writing—original draft. **Jaffer Shah**: Data curation; writing—review and editing. **Hani Aiash**: Writing—review and editing.

## CONFLICT OF INTEREST

The authors declare no conflict of interest. The lead author affirms that this manuscript is an honest, accurate, and transparent account of the study being reported; that no important aspects of the study have been omitted; and that any discrepancies from the study as planned (and, if relevant, registered) have been explained.

## Supporting information

Supplementary information.
