# Supplementary material for: Association of familial Mediterranean fever and epicardial adipose tissue: A systematic review and meta‐analysis
Source: Health Sci Rep. 2022 Jun 13;5(4):e693. doi: 10.1002/hsr2.693 (PMC9193962; doi:10.1002/hsr2.693)
Supplement: Supplementary file 1 — Supplementary information. [file HSR2-5-e693-s001.docx]

Supplementary material

Detailed databases search strategies and date consulted

1.Pubmed

Date consulted : 28 march 2022

PubMed: ("Familial Mediterranean Fever" OR "Familial Paroxysmal Polyserositis" OR "Periodic Disease" OR "Periodic Peritonitis" OR "Recurrent Polyserositis" ) AND ( “epicardial adipose tissue " OR " lipids " )

2. Ovid:

Date consulted : 28 march 2022

("Familial Mediterranean Fever" OR "Familial Paroxysmal Polyserositis" OR "Periodic Disease" OR "Periodic Peritonitis" OR "Recurrent Polyserositis" ) AND ( “epicardial adipose tissue " OR " lipids " )

3. WOS:

Date consulted : 28 march 2022

("Familial Mediterranean Fever" OR "Familial Paroxysmal Polyserositis" OR "Periodic Disease" OR "Periodic Peritonitis" OR "Recurrent Polyserositis" ) AND ( “epicardial adipose tissue " OR " lipids " )

4. Scopus:

Date consulted: 28 march 2022

"familial Mediterranean fever" AND "epicardial adipose tissue"

5.Embase

Date consulted : 28 march 2022

("Familial Mediterranean Fever" OR "Familial Paroxysmal Polyserositis" OR "Periodic Disease" OR "Periodic Peritonitis" OR "Recurrent Polyserositis" ) AND ( “epicardial adipose tissue " OR " lipids " )

6- Open Grey, Lilacs, and Proquest databases

Date consulted : 2 April 2022

"familial Mediterranean fever" AND "epicardial adipose tissue"
